# Gross Cleanliness in Surgery

**Published:** 1904-11-05

**Authors:** 


### GROSS CLEANLINESS IN SURGERY.

There is an idea of gross cleanliness, the application of which, thinks Dr. Robert T. Morris,[1] is full of harmful consequences. An outgrowth of modern methods of wound treatment is the belief that wound discharges in general are unclean and must be removed. Trained nurses and dressers, he complains, are so imbued with this idea that it is difficult to teach them the advantages of skilful neglect. In a healthy granulating wound, for example, new epithelium will be spreading from the skin around the wound. If the pus be removed by swabbing or washed away with an antiseptic solution the delicate new epithelium is damaged or destroyed and repair is delayed, with the result that an undue amount of connective tissue will be formed. A healing wound must therefore be carefully protected against cleaning, and also it should have some such material as silver foil, Cargile membrane, or oiled silk, between the raw surface and the absorbent dressing, so that when the dressing is removed the newly-formed epithelium is not torn away. The same reasoning is capable of practical application in the operative treatment of peritoneal infections; and Dr. Morris deprecates the exhibition of sustained efforts to remove all septic matter from the peritoneum by washing and wiping, and by putting in masses of gauze for drainage. Such expedients deprive the patient of much of his natural power of resistance. It is very easy indeed, concludes Dr. Morris, to produce death in such cases by conscientious attention.

[1] Med. Rec., Oct. 1, 1904.

